# Prevalence Screening for Familial Optic Disc Drusen: A Cross-Sectional Study

**DOI:** 10.1080/01658107.2024.2372624

**Published:** 2024-07-17

**Authors:** Alvilda H. Steensberg, Lasse Malmqvist, Mette Bertelsen, Karen Grønskov, Steffen Hamann

**Affiliations:** a Department of Ophthalmology, Copenhagen University Hospital – Rigshospitalet, Glostrup, Denmark; b Department of Clinical Genetics, Copenhagen University Hospital–Rigshospitalet, Copenhagen, Denmark; c Department of Clinical Medicine, University of Copenhagen, Copenhagen, Denmark

**Keywords:** Optic disc drusen inheritance, optic disc drusen, optic nerve head drusen, EDI-OCT, genetics, heredity

## Abstract

Optic disc drusen (ODD) may present in multiple individuals and generations within a family, indicating hereditary predisposition. Individuals are often unaware of their ODD, and consequently, the prevalence of ODD within families remains largely unknown. The aim of this study was to estimate the prevalence and consider the inheritance pattern of familial ODD and to investigate ODD-related symptoms and their association to ODD location and size. In this cross-sectional study, 22 ODD patients, aged 10 years or older, were included. Of these, 13 ODD probands had 24 family members participating. The presence, size, and anatomical location of ODDs and the presence of hyperreflective lines were ascertained using enhanced depth imaging optical coherence tomography (EDI-OCT) scan. Visual symptoms were ascertained, and these were correlated with ODD burden in terms of ODD location and size. Familial ODD was found in eight of the 13 screened families. Hyperreflective lines were present in all individuals with ODD, including both probands and their family members, and in seven out of the 14 family members without ODD. Visual symptoms were reported in 14–50% and associated significantly with superficial ODD location. We found familial ODD in eight of the 13 screened families. Our results aligned with the previously suggested autosomal dominant inheritance pattern with incomplete penetrance, assuming that hyperreflective lines represent a less expressed form of ODD. This emphasizes that ODD runs in families and supports the hypothesis that genetic factors may contribute to the etiology.

## Introduction

Optic disc drusen (ODD) are calcified deposits in the optic nerve head affecting 2% of the general population.^1,2^ ODD is present in various ways, such as sporadic, linked to other diseases, or manifest within families.^3,4^ Studies reporting families with multiple individuals and generations affected suggest a hereditary predisposition for ODD development.^5,6^ These pedigree studies used diagnostic methods that today are considered suboptimal for identifying smaller and more deeply located ODD. Today, enhanced depth imaging optical coherence tomography (EDI-OCT) of the optic nerve head is considered the gold standard for identifying ODD.^7–9^ With this diagnostic method, not only small and deep ODD can be identified but also hyperreflective lines, prevalent in 3–12% of the population.^10,11^ These lines are suggested to be an indicator of crowding and shown to be more prevalent in some conditions like retinitis pigmentosa or papilledema due to idiopathic intracranial hypertension.^4,12^ Additionally, they are suggested to be potential precursors for ODD development in children.^10^ Alongside the suggested hereditary predisposition for ODD development, the size of the optic nerve scleral canal, where Bruch’s membrane opening can be used as a proxy, has also been proposed as a pathogenic factor.^13–16^ However, studies on this topic have reported contradictory results.^17,18^

Upon examination, most patients with ODD have visual field defects, and ODD is a well-established risk factor for nonarteritic anterior ischemic optic neuropathy (NA-AION).^3,19–21^ Paradoxically, the condition is mainly asymptomatic, and consequently, the individuals are often unaware of their ODD. While families with hereditary ODD have previously been described, the prevalence of familial ODD remains unknown.

The aim of this study was to determine the prevalence of familial ODD by screening family members of ODD patients for the presence of ODD using gold standard diagnostic technology. Additionally, we investigated the potential relationship between several visual symptoms and ODD burden in terms of location and size.

## Methods

### Study population

In this cross-sectional study, patients with an ODD diagnosis and their family members were included. ODD patients who were 10 years or older and who within the past year, as part of being enrolled in another ODD study, had consented to being contacted for potential inclusion in a future research project on ODD, were contacted. The ODD patients aged 18 years or older were requested to ask family members about their interest in participating in the study as well. For the ODD patients aged 10–17 years, their parents were asked to participate. Biological familial relations to the ODD patient included children, grandchildren, siblings, half siblings, parents, and grandparents. Recruitment took place between June 2023 and January 2024 and examinations took place at the Kennedy Centre (Rigshospitalet, Glostrup, Denmark). This project was approved by The Scientific Ethics Committees for the Capital Region of Denmark (H-20027930), and informed consent was obtained after the examiner (A.S.) provided both written and verbal details about the project. The project adhered to the tenets of the Declaration of Helsinki and followed the Strengthening the Reporting of Observational Studies in Epidemiology (STROBE) checklist for cross-sectional studies (Supplemental File 1).

### Data acquisition

At the time of inclusion, OCT scans (Heidelberg Engineering, Heidelberg, Germany) of the optic nerve head according to the Optic Disc Drusen Studies (ODDS) Consortium guidelines^8^ had already been performed on the ODD patients. This included two scan modalities: a dense optic nerve head scan with EDI enabled and 30 µm between each B-scan (97 scans) and a radial circle optic nerve head scan performed in non-EDI mode, with either 6 or 24 scans centered in the middle of the optic disc. New OCT scans were obtained if these prior scans were of inadequate quality and for all included family members.

All ODD patients aged 18 years or older and family members presenting with ODD, responded to four questions regarding specific ODD symptoms: Q1) Have you experienced previous or present obscuration? Q2) Have you experienced dimness of vision? Q3) Have you experienced sensation of visual field defects? Q4) Have you experienced attacks of blindness? These questions were the same as those asked in the study by Lorentzen.^3^

### Analysis of optical coherence tomography data

**Figure 1.**
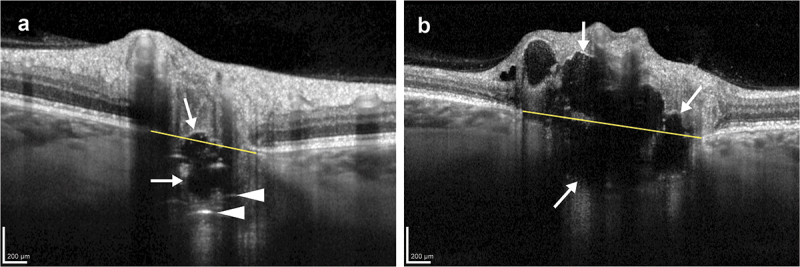
Central sections from dense, enhanced depth imaging optical coherence tomography scans of the optic nerve head. (a) Shows a large and predominantly deep optic disc drusen (arrows) with hyperreflective lines (arrowheads), while (b) shows large, predominantly superficial, and symptom-causing optic disc drusen. Bruch’s membrane opening is visualized (yellow line).

The OCT scans were assessed both by the examiner (A.S.) and specialist (S.H.) using Heidelberg Eye Explorer software (version 1.12.1.0; Heidelberg Engineering). The diagnostic criteria for ODD were defined as hyporeflective elements with a full or partial hyperreflective margin within the optic nerve head.^8^ The ODD were categorized as either large (>200 μm in at least one direction) or small (<200 μm).^22^ Participants were grouped as having ODD if they had at least one ODD, and they were grouped as having large ODD if they had large ODD in at least one eye. The location of the ODD was categorized as superficial or deep, according to whether most of the ODD was located above or below the Bruch’s membrane opening (Figure 1). Prelaminar hyperreflective lines were identified as hyperreflective, intact lines (>50 μm) located in the prelaminar portion of the ONH (Figure 1).^10^ Familial ODD was defined as two or more cases of ODD in one family. The mean Bruch’s membrane opening was determined by averaging the distances between the edges of Bruch’s membrane on either side of the optic disc across six radial disc-centered OCT scans. The data were categorized based on the presence or absence of ODD. For individuals with unilateral ODD, the unaffected eye was excluded from the analysis, as it was hypothesized not to be entirely unaffected and could potentially introduce bias to our results. Missing data from radial circle optic nerve head OCT scan was removed from the analysis.

### Analysis of symptomatology data

The symptoms were analyzed in relation to the size and location of ODD, determined on OCT. In terms of size, patients were categorized as having ‘major’ ODD if both eyes had large ODD and ‘minor’ if at least one eye had small ODD or if the patient had unilateral ODD. Similarly, in terms of location, patients were categorized as having ‘major’ ODD if both eyes had superficial ODD and ‘minor’ if at least one eye had deep ODD or if the patient had unilateral ODD. These categorizations were based on the assumption that eyes with small, deep, or unilateral ODD were less likely to cause symptoms, hence the term ‘minor’. Patients with other potential vision-affecting disease were excluded from the symptomatology analysis.

### Statistics

The study size was chosen to include as many eligible participants, both ODD patients and family members, as possible, from the group of potential participants defined in ‘study population’. The statistical software R (version 2023.09.0) was used for statistical analysis. Continuous variables were presented as median values and interquartile ranges (IQRs). For the analysis of Bruch’s membrane opening, clustering was employed to optimize data inclusion from both eyes.

The Fischer’s exact test was employed to assess the significance of any differences in the proportions of symptoms between the patients with ‘major’ and ‘minor’ ODD. The determined level of statistical significance for all comparisons was *p* > .05.

## Results

**Figure 2.**
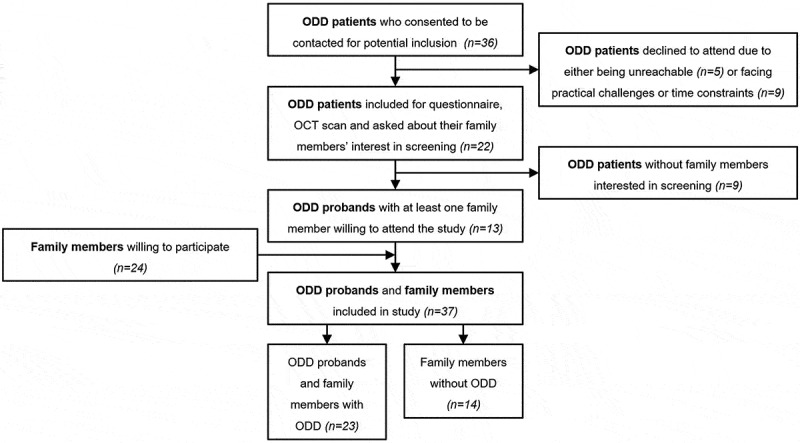
Flowchart illustrating the inclusion of optic disc drusen patients and their families.

**Figure 3.**
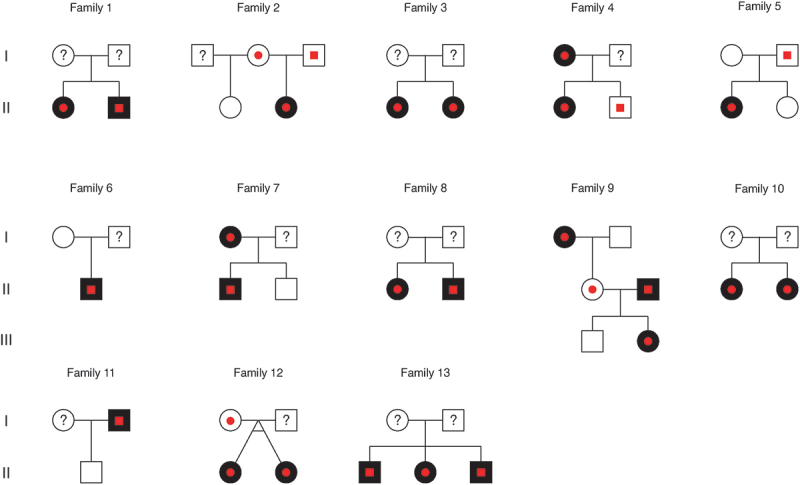
Family pedigrees for the included families, illustrating optic disc drusen affected (black), non-affected (white), hyperreflective lines (red dots) and not examined (question mark) family members.

Out of 36 contacted ODD patients, 22 were included in this study (Figure 2). Out of the 22 initially included ODD patients, 13 probands (59.1%) had at least one family member willing to attend the study, resulting in the inclusion of 13 families. With 24 family members willing to attend the study for screening, the total number ended at 37 participants. Of these, 15 (59%) were male, 22 (41%) were female, and the age ranged from 10 to 84 years (median: 44 years; IQR: 31–61 years). The number of included family members varied between one and five (Figure 3). For family 12, one pair of monozygotic twins under the age of 18 visited the clinic together, both with an ODD diagnosis. While both twins can be considered probands within the family, they are treated as a single proband for the purpose of this study, as they are presented together and belong to the same family. Table 1 shows the demographics and structural findings of the patients and family members in the study.

**Table 1.** Demographics and structural findings in patients and family members with optic disc drusen and family members without optic disc drusen.

	With ODD		Without ODD		*P* Values
	Number	Median (IQR)	Number	Median (IQR)	
Patients, *n* (female %)	24^a^ (64)		14 (50)		
Age, years		46 (23–63)		50 (38–56)	.675^b^
BMO (µm)		1488 (1386–1640)		1520 (1380–1636)	.519^c^

Abbreviations: ODD, Optic Disc Drusen; IQR, Interquartile Range; n, patients; BMO, Bruch’s Membrane Opening.

^a^One additional patient with ODD is included in this table compared to the flow chart (Figure 1). This is because in the flow chart, the twins are defined as a single proband, whereas in the above analysis, data from both twins are included.

^b^Mann-Whitney-Wilcoxon Test.

^c^Two-level hierarchical clustering using linear mixed models with random effects.

**Figure 4.**
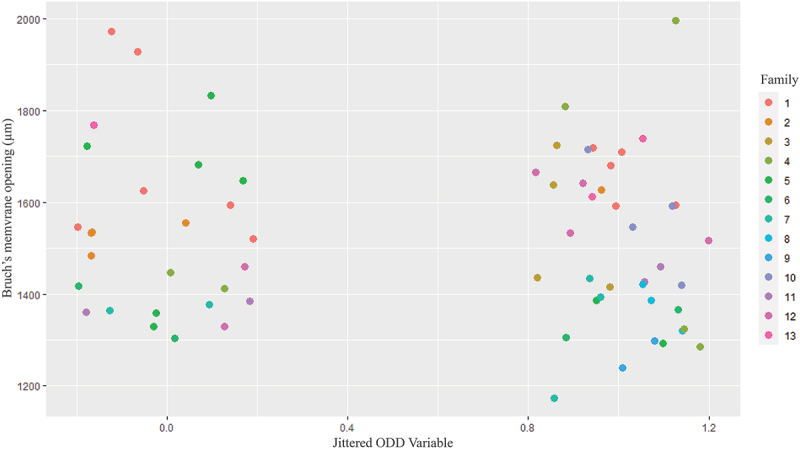
Scatter plot depicting the diameter of Bruch’s membrane opening in family members with and without optic disc drusen, color-coded according to family groups. 0 = No optic disc drusen, 1 = with optic disc drusen.

We found familial ODD in eight of the 13 screened families (61.5%). Out of the 13 probands, 12 (92.3%) showed large ODD, while one showed small ODD (7.7%), and 12 were bilateral (92.3%) and one unilateral (7.7%). Out of the ten family members with ODD, three showed large ODD, while seven showed small ODD and five were bilateral and five unilateral. Hyperreflective lines were present in all individuals with ODD, including both probands and their family members, and in seven out of the 14 family members without ODD corresponding to 50% (Figure 3). For the Bruch’s membrane opening measurements, five eyes were excluded due to missing data and six eyes due to unilateral ODD. There was no significant difference in Bruch’s membrane opening between patients with and without ODD (Table 1). This result is also apparent in the visualization of Bruch’s membrane opening among family members with and without ODD, with each family differentiated by distinct color coding (Figure 4).

Of 22 patients asked for symptoms, four were excluded due to NA-AION, and four due to other potential vision-affecting disease (migraine with aura, multiple sclerosis, internal carotid dissection, and traumatic brain injury). The answers to the questions are presented in Table 2.

**Table 2.** Symptomatology for optic disc drusen patients.

	Prevalence (%)	‘Major’ ODD	*P* values, Fisher’s exact test
Q1) Have you experienced previous or present obscurations?	50	Location	.012
		Size	.002
Q2) Have you experienced dimness of vision?	14	Location	.013
		Size	.078
Q3) Have you experienced sensation of visual field defects?	27	Location	.025
		Size	.056
Q4) Have you experienced attacks of blindness?	0	Location	NA
		Size	NA

Abbreviations: ODD, optic disc drusen; NA, not applicable.

## Discussion

In this study, we found familial ODD in eight of the 13 screened families. The majority of ODD in probands were large, accounting for 92%, compared to 30% in family members.Symptomatology including transient visual obscurations, dimness of vision, and a sensation of visual field defects were found in 50%, 14%, and 27%, respectively.

The prevalence of familial ODD was surprisingly high, keeping in mind that in more than half of the screened families we only screened one family member. Prior to this study, the prevalence of familial ODD was unknown, as existing studies were case reports, case series, and investigations of already identified ODD families, indicating its relative rarity.

Determining the inheritance pattern of ODD in these families was not possible due to the limited number of examined family members and generations. However, autosomal dominant inheritance with incomplete penetrance has been suggested by Lorentzen et al.^5^ This study does not contradict this hypothesis, especially if hyperreflective lines are included in the analysis, under the assumption that they represent a less expressed form of ODD. However, other inheritance patterns cannot be excluded.

The method we used for ODD detection was EDI-OCT, the diagnostic gold standard method for identifying ODD.^9^ When comparing our results to previous studies by Lorentzen and by Antcliff & Spalton,^5,6^ it appears that if today’s methodology had been available at that time, more cases of ODD within the examined families would likely have been identified. With EDI-OCT, hyperreflective lines were observed in 50% of the family members without ODD. Being able to identify hyperreflective lines, small and deep ODD, and after including these into the analysis, it is plausible that the suggested incomplete penetrance more likely reflects varying expression.

When considering ODD as a genetic disease, it is plausible that factors responsible for ODD development are present in both eyes. However, the presence of unilateral ODD suggests potential involvement of local ocular factors and a multifactorial etiology. Often, hyperreflective lines are observed in the fellow ‘healthy’ eye, indicating an unfulfilled potential or a predisposition for ODD development. We hypothesize a multifactorial etiology for ODD, with a strong genetic risk factor, following an autosomal dominant inheritance pattern; however, genetic heterogeneity is also a possibility following other inheritance patterns.

In this study, we found that transient visual obscuration, dimness of vision, and a sensation of visual field defects were found in 50%, 14%, and 27%, respectively. All symptoms were significantly associated with ‘major’ ODD burden in terms of location. The proportion of reported symptoms is higher compared to the findings in the study by Lorentzen,^3^ where both obscuration and dimness of vision were reported in 9% of patients. This difference in symptom prevalence may be partly attributed to different symptomatology assessment methods between the studies, as the study by Lorentzen relied on either questioning the patients or by reviewing the earlier case records available. In this study, all patients were directly questioned, potentially leading to a higher rate of symptom reporting compared to retrospectively searching in case records.

The utilization of EDI-OCT is a strength of the study, as this method identifies ODD of all sizes and locations. Limitations include the relatively small sample size, both the number of probands and of family members and generations. It is also important to note that the majority of the ODD patients exhibited large and bilateral ODD, causing sampling bias. This is particularly noteworthy in the context of symptomatology, given the high prevalence of ODD symptoms. Therefore, these results might mainly apply to patients with large ODD. It is important to underline, that we investigated the sensation of visual field defects, and do not know if the patients had actual visual field defects as measured with perimetry. A study dedicated to ODD symptomatology, including visual fields and ODD characteristics, is warranted.

We found familial ODD in eight of the 13 screened families, suggesting a notably high familial ODD prevalence. It is important to note that in more than half of the screened families, only one family member was screened, potentially leading to an underestimation of this prevalence.

Future studies should strive toward including a greater number of family members and generations to get a more accurate estimation of the true prevalence of familial ODD. Further genetic studies of families with ODD may provide insight into the importance of genetic elements in the causation of ODD.

## Supplementary Material

Supplemental Material
